# Polar bears experience skeletal muscle atrophy in response to food deprivation and reduced activity in winter and summer

**DOI:** 10.1093/conphys/cox049

**Published:** 2017-08-09

**Authors:** John P. Whiteman, Henry J. Harlow, George M. Durner, Eric V. Regehr, Bryan C. Rourke, Manuel Robles, Steven C. Amstrup, Merav Ben-David

**Affiliations:** 1 Program in Ecology, University of Wyoming, 1000 E. University Avenue, Laramie, WY 82071, USA; 2 Department of Zoology and Physiology, University of Wyoming, 1000 E. University Avenue, Laramie, WY 82071, USA; 3 U.S. Geological Survey, Alaska Science Center, 4210 University Drive, Anchorage, AK 99508, USA; 4 Marine Mammals Management, U.S. Fish and Wildlife Service, 1011 East Tudor Road, Anchorage, AK 99503, USA; 5 Current: Polar Science Center, Applied Physics Laboratory, University of Washington, 1013 NE 40th Street, Seattle, WA 98105, USA; 6 Department of Biological Sciences, California State University, 1250 Bellflower Blvd, Long Beach, CA 90840, USA; 7 Polar Bears International, P.O. Box 3008, Bozeman, MT 59772, USA

**Keywords:** Fasting, myosin heavy-chain isoforms, protein concentration, *Ursus maritimus*

## Abstract

When reducing activity and using stored energy during seasonal food shortages, animals risk degradation of skeletal muscles, although some species avoid or minimize the resulting atrophy while experiencing these conditions during hibernation. Polar bears may be food deprived and relatively inactive during winter (when pregnant females hibernate and hunting success declines for other demographic groups) as well as summer (when sea ice retreats from key foraging habitats). We investigated muscle atrophy in samples of biceps femoris collected from free-ranging polar bears in the Southern Beaufort Sea (SBS) throughout their annual cycle. Atrophy was most pronounced in April–May as a result of food deprivation during the previous winter, with muscles exhibiting reduced protein concentration, increased water content, and lower creatine kinase mRNA. These animals increased feeding and activity in spring (when seal prey becomes more available), initiating a period of muscle recovery. During the following ice melt of late summer, ~30% of SBS bears abandon retreating sea ice for land; in August, these ‘shore’ bears exhibited no muscle atrophy, indicating that they had fully recovered from winter food deprivation. These individuals subsequently scavenged whale carcasses deposited by humans and by October, had retained good muscle condition. In contrast, ~70% of SBS bears follow the ice north in late summer, into deep water with less prey. These ‘ice’ bears fast; by October, they exhibited muscle protein loss and rapid changes in myosin heavy-chain isoforms in response to reduced activity. These findings indicate that, unlike other bears during winter hibernation, polar bears without food in summer cannot mitigate atrophy. Consequently, prolonged summer fasting resulting from climate change-induced ice loss creates a risk of greater muscle atrophy and reduced abilities to travel and hunt.

## Teaser

Polar bears experience skeletal muscle atrophy during winter whether they hibernate or not. Bears spending summer on sea ice in the Arctic basin undergo a second period of atrophy caused by fasting, whereas bears with shore access to adequate, alternative food do not. Summer atrophy may influence fitness.

## Introduction

In response to seasonal declines in food availability, many animal species reduce their activity and catabolize endogenous tissue for energy (Wang *et al.*, 2006; McCue, 2010). Reduced activity decreases skeletal muscle loading and neural activation, potentially causing atrophy that is characterized by diminished muscle mass and strength (Haddad *et al.*, 2003a; Hanson *et al.*, 2013; Phillips *et al.*, 2009). Fasting-induced catabolism can also cause muscle atrophy (Wing and Goldberg, 1993) and usually includes three phases: animals primarily oxidize stored carbohydrates in the first phase and stored lipids in the second (McCue, 2010). In both of these phases, some endogenous protein is degraded to supply amino acids for biosynthetic processes involved in cell replacement and tissue repair (Lee and Davis, 1979). This obligatory protein catabolism, in addition to the use of amino acids as oxidative substrate, eventually causes atrophy-related pathologies (Jagoe *et al.*, 2002; Allen *et al.*, 2010). Once protein loss becomes extensive, animals enter the third phase of fasting, which, unless feeding resumes, culminates in death (Castellini and Rea, 1992).

Many of the cellular and molecular changes that occur during atrophy in skeletal muscle are similar regardless of whether they are caused by reduced activity or food deprivation. As muscle protein concentration declines, water content tends to increase (McCue, 2010), often concurrent with no change or even an increase in the amount of DNA per unit mass (Heymsfield *et al.*, 1982; Haddad *et al.*, 2003b). Processes responsible for growth, energy storage, and oxygen delivery in muscle tissue are altered, often through changes in mRNA expression (Jaynes *et al.*, 1986; Pilegaard *et al.*, 2003). For example, use of phosphocreatine, a temporary energy store in muscle, likely declines during atrophy (Bogdanis, 2012); accordingly, gene expression for creatine kinase (CK), the enzyme responsible for its synthesis, is reduced (Washabaugh *et al.*, 2001; Jagoe *et al.*, 2002). Simultaneously, muscle growth is slowed via increased expression of mRNA for the growth-inhibiting transcription factor myostatin (MYO; Wehling *et al.*, 2000; Allen *et al.*, 2010). Hypoxia-inducible factor (HIF) enhances oxygen availability and may act to reduce aerobic oxidation. As capillary density and energy stores often decline during atrophy, HIF mRNA may rise (Cornachione *et al.*, 2011; Soñanez-Organis *et al.*, 2013).

Other characteristics of atrophy differ depending on whether they were induced by food deprivation or inactivity. The mRNA of the growth-stimulating hormone, insulin-like growth factor (IGF), declines during food deprivation (VandeHaar *et al.*, 1991). However, during inactivity IGF mRNA may increase (Haddad *et al.*, 2003b), decrease (Awede *et al.*, 1999), or remain unchanged (Yang *et al.*, 1997). Muscle carbon-to-nitrogen ratio (CN), an index of myocellular lipid stores (Post *et al.*, 2007), declines during food deprivation but rises during inactivity (Manini *et al.*, 2007). Muscle phenotype primarily reflects use and activation, as inactivity causes myocytes to exhibit decreased fiber cross-sectional area (Allen *et al.*, 1995), to transition from slow oxidative (SO) to fast-glycolytic (FG) fiber types (Pette, 2003), and to decrease expression of the type I isoform of myosin heavy-chain protein (MyHC) while increasing that of type IIa or IIx (Haddad *et al.*, 2003a).

Hibernators undergo months of simultaneous fasting and inactivity during winter, yet have remarkable abilities to reduce or avoid atrophy (Lohuis *et al.*, 2007b; Cotton and Harlow, 2010). The ability of bears (family *Ursidae*), the largest hibernators, to avoid atrophy is notable because unlike smaller animals (e.g. squirrels, family *Sciuridae*), bears maintain near-normal body temperature during hibernation (30–35°C) and remain alert and capable of quick movement when disturbed (Harlow, 2012). Black (*Ursus americanus*) and brown bears (*Ursus arctos*) are well-adapted to minimize muscle atrophy (Tinker *et al.*, 1998; Lohuis *et al.*, 2007a; Hershey *et al.*, 2008). These adaptations include catabolism of sources of endogenous protein other than skeletal muscle (e.g. connective tissue; Harlow, 2012), reduction of protein breakdown rates, and recycling of >99% of urea nitrogen into new tissue (Barboza *et al.*, 1997). As a result, black bears lose only 23% of their tibialis anterior strength during winter food deprivation and inactivity, as opposed to the 90% loss predicted for humans in the same scenario (Harlow *et al.*, 2001). However, these adaptations are not manifested during food deprivation in summer (Nelson *et al.*, 1975; Barboza *et al.*, 1997).

Across the Arctic, polar bears (*Ursus maritimus*) experience seasonal food deprivation and changes in activity (Watts and Hansen, 1987; Ramsay *et al.*, 1991; Whiteman, 2014). Similar to some other Ursids, pregnant females hibernate, give birth, and nurse in maternal dens from approximately November through March. Males and non-pregnant females remain active during this period although their hunting success is reduced (Stirling and Oritsland, 1995), probably because the main prey of polar bears, ringed seals (*Pusa hispida*), spend little time on the sea ice surface in winter (Kelly *et al.*, 2010). Beginning in April, ringed seals give birth and naïve pups become available while adults increase their surface time for nursing and molting (Kelly *et al.*, 2010), increasing their vulnerability to predation (Stirling and Archibald, 1977; Pilfold *et al.*, 2013). Mother polar bears and their new cubs emerge from dens, and all bears become highly active and hyperphagic for April–July (Ramsay and Stirling, 1988; Whiteman, 2014).

Annual ice melt then peaks during August–October, forcing some polar bears to fast again. In the Southern Beaufort Sea (SBS), one of 19 subpopulations (Obbard *et al.*, 2010), ~70% of individuals follow the retreating sea ice as it recedes north beyond the shallow and productive continental shelf waters, while ~30% come to shore (Atwood *et al.*, 2016; Pongracz and Derocher, 2017). Bears that follow the ice retreat become food deprived (Whiteman, 2014) because the ice withdraws to deep water that probably has few ringed seals (Harwood and Stirling, 1992; Harwood *et al.*, 2012). In this subpopulation, most bears that spend summer on shore maintain their body condition by scavenging bowhead whale (*Balaena mysticetus*) carcasses deposited on the beach after Iñupiat harvest (Miller *et al.*, 2015; Whiteman, 2014). Nutritionally rich foods on land, however, are generally scarce in other parts of the Arctic (Rode *et al.*, 2015). As a result, bears on shore in other subpopulations, such as Western Hudson Bay, lose ~0.9 kg of body mass per day during summer (Atkinson *et al.*, 1996; Pilfold *et al.*, 2016). In the SBS subpopulation, individuals on shore and on sea ice also concurrently decrease their activity in this season (Whiteman *et al.*, 2015); thus, some bears experience food deprivation and reduced activity during both winter and summer (Table 1). These individuals potentially experience muscle atrophy in both seasons, including reduced strength and fatigue resistance (Bogdanis, 2012), which could make it more difficult to travel and hunt. Possible seasonal atrophy is particularly important because sea ice loss is extending fasting periods and has been linked to declines in survival and abundance (Regehr *et al.*, 2010; Bromaghin *et al.*, 2015; Rode *et al.*, 2010). However, muscle atrophy across seasons has not been investigated in polar bears.

**Table 1:** Idealized timeline of the polar bear annual cycle in the southern Beaufort Sea

	November–March	April–June	July	August–October
% time active	Pregnant bears: ≤4%	25%	Transition to shore or ice over deep water	10–25%
	Other bears: 10–25%			
Nutrition	Mostly food deprived	Feeding		Ice: mostly food deprived
				Shore: scavenging whale

Although July is indicated as a time of transition between habitats, this is a continuous process and bears may swim between shore and ice repeatedly in a given year (Durner *et al.*, 2011; Pagano *et al.*, 2012). Values for time spent active are approximations (Messier *et al.*, 1992; Ferguson *et al.*, 2001; Whiteman *et al.*, 2015).

In a recent study, we examined the nutritional status and activity patterns of bears in the SBS after winter food deprivation and during summer fasting (Whiteman, 2014; Whiteman *et al.*, 2015). Here, in a companion study, we augment those findings by evaluating muscle samples from the same individuals and testing predictions that: (1) bears on the sea ice over the continental shelf in April–May are recovering from atrophy induced by reduced activity and food deprivation during the previous winter; (2) bears on shore in August demonstrate no atrophy, as a result of prior high activity and hyperphagy during spring and early summer; (3) bears on shore in October exhibit little to moderate atrophy because in the months prior to sampling they had experienced competing influences of reduced activity and feeding on whale; and (4) bears on sea ice over deep water in October show moderate to substantial atrophy as a result of their reduced activity and fasting over the previous summer. We expected that atrophy would be more severe in 2008 than in 2009 because of greater ice melt that year (NSIDC, 2016; Fig. S1). Also, for April–May bears, we predicted that females that hibernated during the preceding winter (i.e. those accompanied by cubs-of-the-year in spring) would exhibit more atrophy than females that did not, because hibernation includes inactivity and complete fasting in maternal dens for up to 7 months.

We evaluated atrophy in samples of biceps femoris muscle collected from individuals during each season. In addition, we tested two predictions using pooled data from all individuals: first, that muscle protein is positively related to serum albumin, because both are potentially important reservoirs of labile protein; and second, that expression of type I MyHC isoform declines in response to lower activity and movement rate. Because muscle responds variably to changes in functional demands over different time periods (Chacon-Cabrera *et al.*, 2016), we tested the influence of activity and movement rate over three different time periods prior to sampling: 43 days (the longest period for which data was available), 28 days (moderate duration), and 5 days (short duration, but long enough for significant changes to occur; Goldspink *et al.*, 1986).

## Materials and methods

### Captures and field data

**Figure 1:**
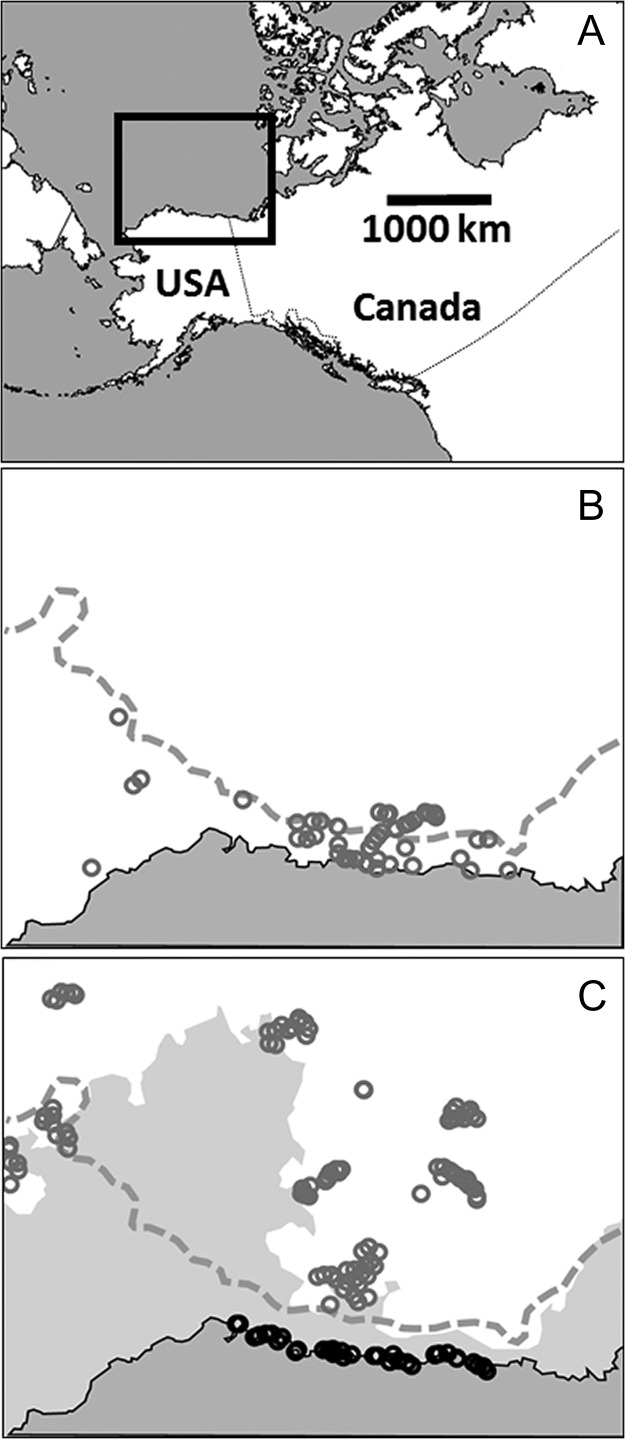
Maps of the study area in the Arctic. (**A**) The Beaufort Sea region (boxed). (**B**) The Beaufort Sea region, Alaskan (USA) coast (dark gray), 300 m bathymetric contour (dashed gray line), and distribution of sea ice (white) as of 11 May 2009. Gray circles are locations (estimated error <250m) from 24 polar bears over the previous week. (**C**) The same area with distribution of sea ice as of 31 August 2009. Circles are locations of 31 polar bears over the previous week, exemplifying ‘ice’ (gray) and ‘shore’ (black) individuals. Ice data from National Snow and Ice Data Center (https://nsidc.org/).

We sampled bears via helicopter captures between Barrow, Alaska (USA), and the USA–Canada border, on the shoreline and ≤150 km offshore on coastal sea ice when present. In October 2009, we also sampled bears on the sea ice between 70–79°N and 132–170°W, from the US Coast Guard Cutter *Polar Sea* (Fig. 1). The study area was covered by less sea ice in 2008 than in 2009 during May–September (NSIDC, 2016; Fig. S1).

We immobilized bears with a mixture of tiletamine hydrochloride and zolazepam hydrochloride (estimated doses of 4–10 mg/kg of body mass; Telazol, Warner-Lambert Co., Morris Plains, NJ, USA) delivered in projectile syringes fired from a gun (Stirling *et al.*, 1989). We monitored bear rectal temperature and respiration rate during immobilization. For newly captured adults, age was determined by counting cementum annuli in extracted vestigial premolars (Calvert and Ramsay, 1998). All bears were uniquely marked with a lip tattoo and ear tags.

We fitted a subset of bears with location transmitters glued to the fur between the shoulder blades (*N* = 2 bears; MK-10, Wildlife Computers, Redmond, WA, USA), attached via ear-tags (*N* = 11; SPOT, Wildlife Computers), or mounted on collars (*N* = 22; TGW-3680 and TGW-4689H; Telonics, Mesa, AZ, USA). Transmitters were primarily used to locate bears for re-capture. In addition, for individuals with GPS transmitters (estimated error <31 m; D’Eon *et al.*, 2002) we calculated movement rate as distance traveled divided by time, using sequential, hourly locations. Also, attached to collars were loggers that recorded either the number of seconds of activity per half hour (*N* = 14; TGW-4689H, Telonics) or the activity intensity every 2 min (a unitless index of acceleration; *N* = 22; Actiwatch, Mini-Mitter Respironics, Bend, OR, USA). We calculated the percent of time a bear spent active as seconds of activity per half hour (which were measured directly or calculated from activity intensity with an equation derived from data collected from bears wearing both logger types; Whiteman *et al.*, 2015).

We collected blood samples from a femoral vessel in vacutainers^®^, centrifuged them at 2000 RCF for 10 min, and siphoned serum for measurement of albumin with a veterinary diagnostic analyzer (VS2 VetScan; Abaxis Inc., Union City, CA, USA). All capture and sampling procedures were approved by institutional animal care and use committees at the University of Wyoming and US Geological Survey Alaska Science Center, and permitted by the US Fish and Wildlife Service (Permit #MA690038).

### Muscle sampling and analyses

With the bear laying prone, we externally rotated the hindlimbs and flexed the knee to ~90°. We marked a location halfway between the knee and the greater trochanter of the femur, and halfway between the femur and distal edge of the upper hindlimb (contralateral limb at re-capture). We shaved an area 5 cm × 10 cm, injected 1 ml of xylocaine into the subcutaneous tissue, scrubbed the skin and surrounding fur with povidone-iodine, then used sterile surgical equipment to incise the skin and adipose tissue. We collected ~500 mg of superficial muscle then closed the adipose tissue (continuous stitches, 00 gauge suture) and skin (interrupted stitches, 0 gauge). Muscle samples were divided into two portions. The first was sealed in foil and a plastic bag and immediately stored in liquid nitrogen for subsequent assays, and the second was embedded in a medium (Tissue-Tek; Sakura Finetek, Torrance, CA, USA) for sectioning and flash-frozen in isopentane chilled with liquid nitrogen. After the completion of field work, samples were assayed and sectioned at the same time.

The tissue sealed in foil and plastic was divided into two subsamples. The first subsample was freeze-dried for ≥48 hours and mass loss was converted to water content (% mass). We then homogenized the dry subsamples in 40:1 (v:w) phosphate-buffered saline (28 372; Thermo Scientific, Waltham, MA, USA) and measured the protein content using a microplate protocol for the Bradford assay with bovine serum albumin as a standard (23 236; Thermo Scientific). Incomplete homogenization occasionally yielded anomalously low values, thus each subsample was divided into four smaller pieces which were independently assayed. We then calculated a mean protein content, discarded results differing by >10% from that mean, and re-calculated a final mean. To measure CN ratio, subsamples were homogenized to powder with metal scissors and spatulas, loaded into tin capsules, and analyzed at the University of Wyoming Stable Isotope Facility for percent carbon and percent nitrogen with a Costech ECS elemental analyzer (Costech Analytical Technologies, Valencia, CA, USA) attached to a continuous flow mass spectrometer. Stable isotope measurements are reported elsewhere (Whiteman, 2014).

The second subsample was used to identify MyHC protein isoforms via SDS-PAGE electrophoresis following Rourke *et al.* (2006). Frozen samples were homogenized with a glass pestle and tube held in an ice slurry, then suspended in 19 vol. of ice-cold protein buffer (250 mmol l^−1^ sucrose, 100 mmol l^−1^ KCl, 5 mmol l^−1^ EDTA). The homogenate was added to sample buffer (5% β-mercaptoethanol, 100 mM Tris-base, 5% glycerol, 4% SDS and 1% bromphenol blue) and heated to 95°C for 2 min. Approximately 1 mg of total protein was loaded into SDS-PAGE vertical slab gels (CBC Scientific, Del Mar, CA, USA). Gels were run for 24 h at 4°C and a constant voltage of 275 V. Two isoforms were identified after silver stain (Bio-Rad, Hercules, CA, USA). Gels were digitally photographed (Canon, Melville, NY, USA) and analyzed for relative MyHC isoform percentages by densitometry (ImageQuant, Molecular Dynamics, Sunnyvale, CA, USA).

To quantify muscle DNA concentration, we used the homogenate from SDS-PAGE analysis for fluorometric measurements using Hoechst stain. DNA standards of known concentration were created using salmon sperm (Life Technologies, Carlsbad, CA, USA). Ten microliter of muscle homogenate was added to assay buffer containing Tris and EDTA, incubated with Hoeschst stain, and read on a fluorescent microplate reader while excited at 350 nm. Concentration of DNA was corrected per μg of total protein, assayed separately from the Bradford assay; protein was measured in triplicate on a microplate spectrophotometer (BioTek, Winooski, VT, USA) using a commercially available kit (Bio-Rad, Hercules, CA, USA) and IgG as standard.

To quantify mRNA of CK, MYO, IGF, and HIF, we used a 15–20 mg piece of the second subsample of muscle. Tissue was homogenized at high speed with a 5 mm metal grinder in TriReagent (Molecular Research Center, Cincinnati, OH, USA) for extraction of total RNA. Phase separation was initiated by addition of BCP reagent (Molecular Research Center), and a fixed volume of the aqueous portion was withdrawn. Pellets were precipitated with isopropanol then rinsed twice with 75% ethanol. The RNA pellets were dried in an evaporator and solubilized in 20 ml of DNase/RNase-free water. Absorbance was read on a UV microplate (BioTek, Winooski, VT, USA) at 260 and 280 nm.

Primers for the genes of CK, MYO, IGF and HIF were created by accessing GenBank information for mammals and accumulating available sequences for each gene of interest. Alignments of multiple species for a single gene were used to identify two or more 20–30 bp sections of identity across species. A pair of upper and lower primers was combined in a trial and error fashion until products were obtained in PCR reactions. This approach was successfully used for a study of heart muscle in brown bears (Barrows *et al.*, 2011), closely related to polar bears (Liu *et al.*, 2014), as at the time of assays, polar-bear specific primers were not available. Reactions were carried out using 1 μg of total RNA reverse-transcribed to cDNA (Superscipt II, Invitrogen/Life Technologies, Carlsbad, CA, USA), which was then added to PCR reactions optimized to varying annealing temperature and Mg^2+^ concentration for each gene. Results were standardized to 18s expression as previously described (Barrows *et al.*, 2011).

To measure muscle fiber cross-sectional area, tissue samples embedded in Tissue-Tek were sectioned on a cryostat (−20°C) and allowed to thaw for 15 min, then stained for succinic dehydrogenase with Trizma hydrochloride and base, succinic acid, nicotinamide adenine dinucleotide, and nitrotetrazolium blue chloride (T3253, T1503, S3674, N4505, N6639; Sigma Aldrich, St. Louis, MO, USA). Stain was dark for SO fibers and light for FG fibers; we excluded those of intermediate color. We cover-slipped slides and imaged them at 40× with a digital camera mounted on a microscope then used ImageJ software (Rasband, 2014) to measure cross-sectional areas for samples with ≥50 fibers.

### Statistical analyses

All analyses were done in the R statistical environment (R Core Team, 2015). To evaluate the relationship between muscle protein concentration and serum albumin, we used an *F* test for the fixed effect of protein concentration in a linear mixed effects model (package lme4; Bates *et al.*, 2014) based on the Kenward–Roger approximation (Halekoh and Højsgaard, 2012), and then calculated the marginal *R*^2^.

We used linear mixed effects models (Bates *et al.*, 2014) to test the influence of one random predictor (bear ID; to account for repeated sampling of some individuals) and two fixed predictors (group: April–May ice, August shore, October shore, October ice; year: 2008, 2009) on variables indicating muscle atrophy. For the variables affected by group, we used pairwise comparisons among groups with two-tailed Welch *t*-tests to clarify differences. Because this involved six comparisons for each variable, we applied a sequential Bonferroni correction to *α*-values (Rice, 1989). For variables affected by group and year, we conducted pairwise comparisons among groups in 2009 only.

We used linear regression models to test the relationship between the independent variables of (a) percent of time spent active (from activity loggers attached to collars) and (b) movement rate (from sequential, hourly GPS locations) on the dependent variable of MyHC composition. Data did not include multiple captures of individuals thus bear ID was not included as a predictor. We evaluated models using activity and movement rate calculated over the 5, 28 and 43 days prior to sampling muscle tissue.

For all models, we evaluated residual normality with the Cramér-Von Mises test and quantile plots (sample versus theoretical), and assessed homogeneity of variance with plots of residual versus fitted values. Response variables were square-root transformed as needed to meet assumptions of normality. For the linear mixed effects model of muscle CN ratios, residuals showed moderate departure from normality (i.e. *P* = 0.05) but passed the alternative D’Agostino test (*P* = 0.30) thus we accepted the model. For all models, we established the criteria that a data point may be an outlier if its removal changed significance of results and its residual was the largest in the model. We set *α* = 0.05 except where indicated.

## Results

**Figure 2:**
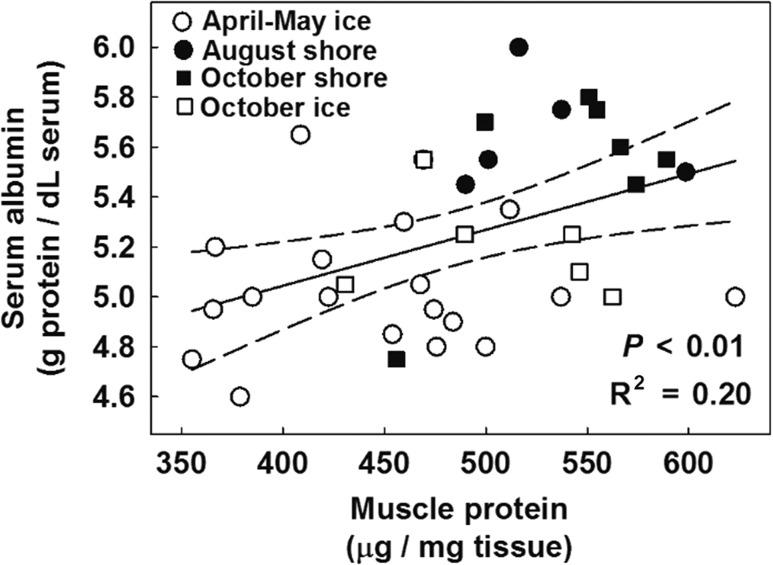
Skeletal muscle protein and serum albumin are positively related in polar bears. Each symbol represents an individual from one of four groups, defined by month and location of capture. All individuals were pooled for the linear regression line (solid; dashed lines are 95% confidence interval). *P* value is for the *F*-test of muscle protein as a predictor in a LME model that included bear ID as a random effect (all individuals pooled). Marginal *R*^2^ value describes the variance explained by this fixed effect.

We collected 54 samples of biceps femoris muscle tissue (50 from females, 4 from males) from 40 individual polar bears (36 females, 4 males). Mean age ± 95% CI for each group was 10 ± 2 years (April–May ice), 10 ± 6 (August shore), 10 ± 3 (October shore), and 9 ± 3 (October ice). Sample sizes for each variable are shown in figures and tables (excluding outliers, presented in Table S1). Groups on shore were sampled in 2008 and 2009: August (combining years, sampling occurred 4–30 August, mean of 17 August) and October (4–28 October, mean 14). Groups on the sea ice were sampled in 2009 only: April–May (11 April–19 May, mean 5 May) and October (3–19 October, mean 9). Combining data from all groups, muscle protein concentration was significantly related to serum albumin (Fig. 2).

### Influence of hibernation on skeletal muscle atrophy

For April–May bears, no variables differed significantly between those that had denned and hibernated during the preceding winter (*N* = 9: females with cubs-of-the-year) and those that likely had not (*N* = 10; Table S2). The latter group included seven bears from demographic classes which typically do not hibernate in winter (one male and six females with yearlings; Amstrup, 2003) and three females without cubs. Of the latter three females, one was too young (4 years) to likely have been pregnant (Lentfer *et al.*, 1980); one had telemetry data which indicated continuous movement during the preceding winter; and one was in estrus and not lactating at capture, suggesting that she did not have cubs recently.

### Skeletal muscle phenotype in different years, seasons and habitats

**Figure 3:**
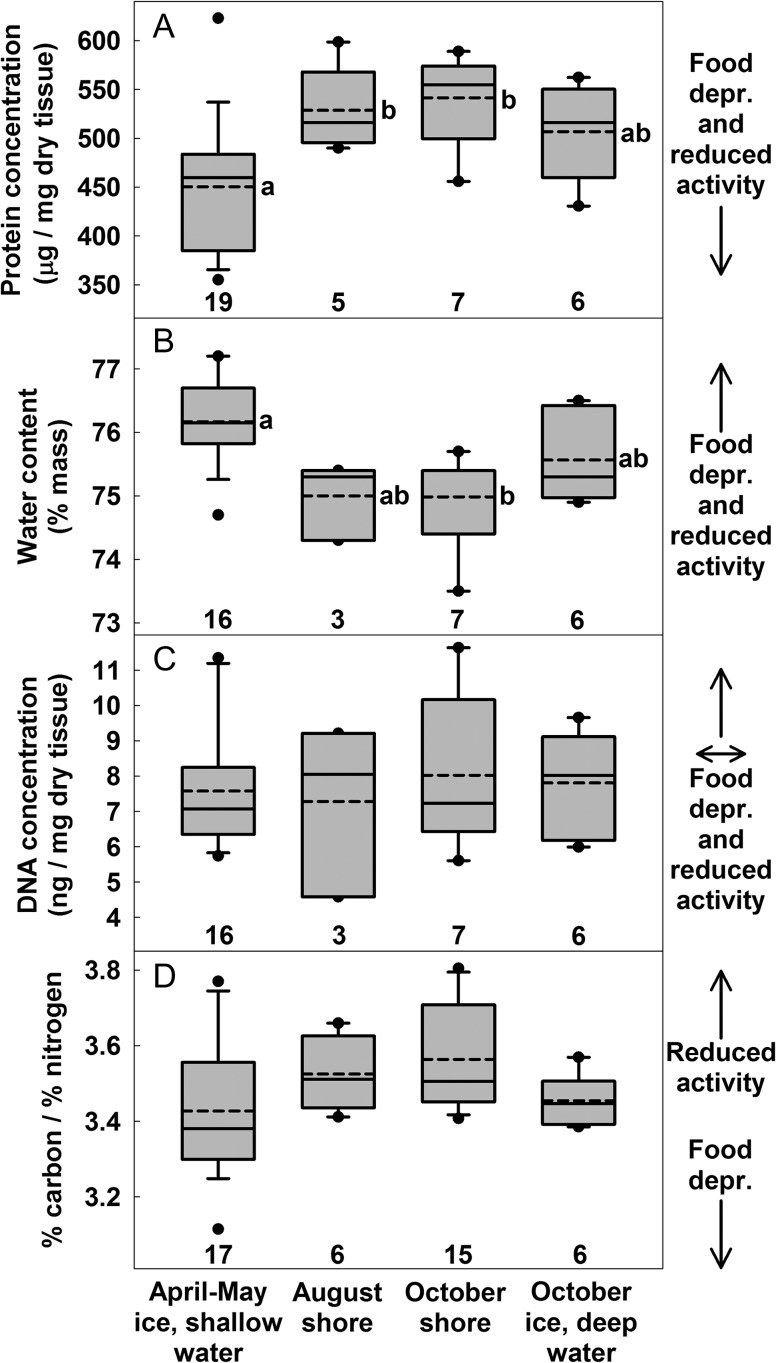
Variables indicating that polar bears exhibited atrophy of the biceps femoris in April–May and in October on sea ice. Dashed and solid lines are mean and median; boxes, bars, and circles are 25th/75th, 10th/90th, 5th/95th percentiles; sample sizes are above *x*-axis. Arrows at the right of panels indicate expected direction of change in response to food deprivation and reduced activity. For (**A**) protein concentration, (**B**) water content and (**C**) DNA concentration, data were collected in 2009. Values were affected by group in A and B (likelihood ratio *P* < 0.01 for both) but not in C (*P* = 0.61). Groups which do not share a lowercase letter differed in pairwise comparisons. (**D**) For CN ratio (% carbon/% nitrogen), data were collected in 2008 and 2009. Values were not affected by year (likelihood ratio *P* = 0.78) thus data from both years were pooled. Values were also not affected by group (likelihood ratio *P* = 0.13) thus no pairwise comparisons were performed.

**Figure 4:**
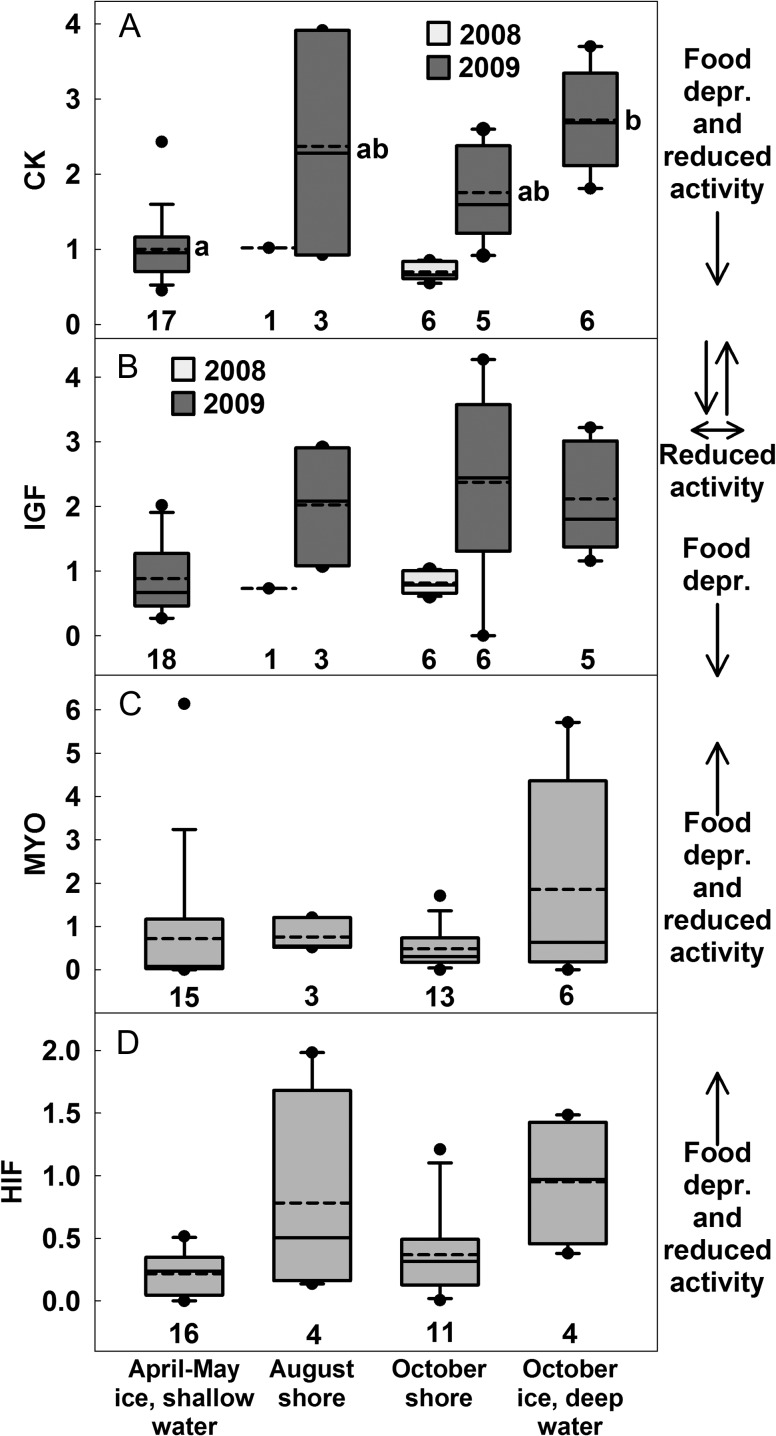
Expression of mRNA (relative to 18s, arbitrary units) of different genes in the biceps femoris of polar bears. Dashed and solid lines are mean and median; boxes, bars, and circles are 25th/75th, 10th/90th, 5th/95th percentiles; sample sizes are above *x*-axis. Arrows at the right of panels indicate expected direction of change in response to food deprivation and reduced activity. All variables were measured in 2008 and 2009. For (**A**) CK, values were affected by year and group (likelihood ratio, *P*_year_ < 0.01, *P*_group_ < 0.01). Pairwise comparisons among groups in 2009 only; groups which do not share a lowercase letter differed (Table S3 lists the *P* values and Bonferroni-adjusted *α* values). For (**B**) IGF, values were affected by year and group (likelihood ratio, *P*_year_ = 0.02, *P*_group_ < 0.01). However, pairwise comparisons were not significant after Bonferroni correction (Table S3). For (**C**) MYO, values were not affected by year or group (likelihood ratio, *P*_year_ = 0.67, *P*_group_ = 0.27) and years were pooled. For (**D**) HIF, values were not affected by year, but were affected by group (likelihood ratio, *P*_year_ = 0.73, *P*_group_ < 0.01), although pairwise comparisons were not significant (see Table S3).

Muscle protein concentration and water content suggested that the greatest atrophy occurred over winter, with the effects detected in bears captured in April–May. Also, based on these variables measured in bears captured in October, moderate atrophy appeared to occur for individuals on the sea ice during late summer and fall (Fig. 3A and B; *P* values and Bonferroni-adjusted *α* values for all group comparisons are listed in Table S3). We could not test the effect of year on protein concentration and water content because these variables were only measured in samples from 2009. DNA concentration and CN ratio did not significantly differ among groups or between years (Fig. 3C and D). Greater atrophy in 2008 than in 2009 for bears on shore in August and October was suggested by reduced mRNA expression of CK and IGF in 2008 (Fig. 4A and B). These two variables also suggested that among 2009 groups only, atrophy was greatest for the April–May bears on the sea ice, although after Bonferroni corrections, group comparisons were only significant for CK. In contrast, mRNA of MYO (Fig. 4C) and HIF (Fig. 4D) did not differ between years. MYO was not affected by group; HIF exhibited an effect, although no subsequent pairwise comparisons were significant.

**Figure 5:**
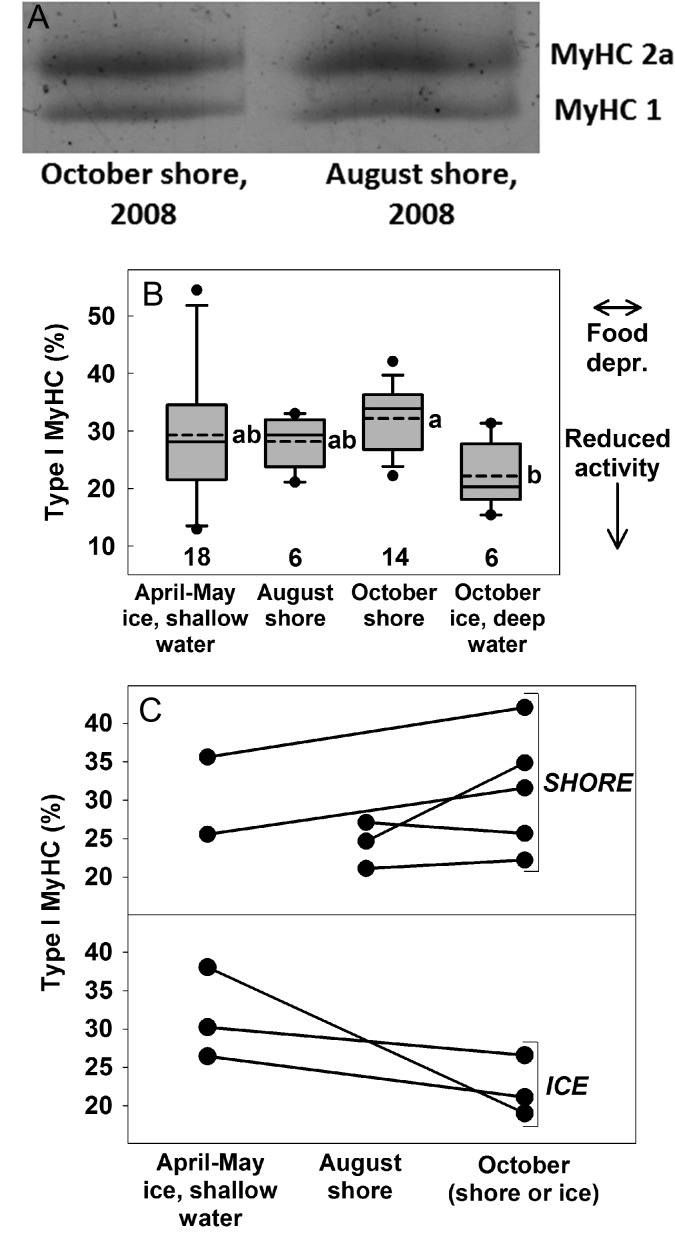
Composition of MyHC isoforms in the biceps femoris of polar bears. (**A**) SDS-PAGE gel illustrating the two MyHC isoforms observed in two polar bears. (**B**) For % type I MyHC isoforms, dashed and solid lines are mean and median; boxes, bars, and circles are 25th/75th, 10th/90th, 5th/95th percentiles; sample sizes are above *x*-axis. Arrows at the right indicate expected direction of change in response to food deprivation and reduced activity. Data were collected in 2008 and 2009. Values were unaffected by year, but differed by group (likelihood ratio, *P*_year_ = 0.12, *P*_group_ = 0.01). Data were pooled between years, and groups which do not share a lowercase letter differed in post-hoc pairwise comparisons (Table S3). (**C**) The % type I MyHC isoforms from a subset of bears in panel A. Each line connects values from an individual bear that was sampled twice: at capture in either spring or August, and at re-capture in October either on shore (top) or ice (bottom).

**Figure 6:**
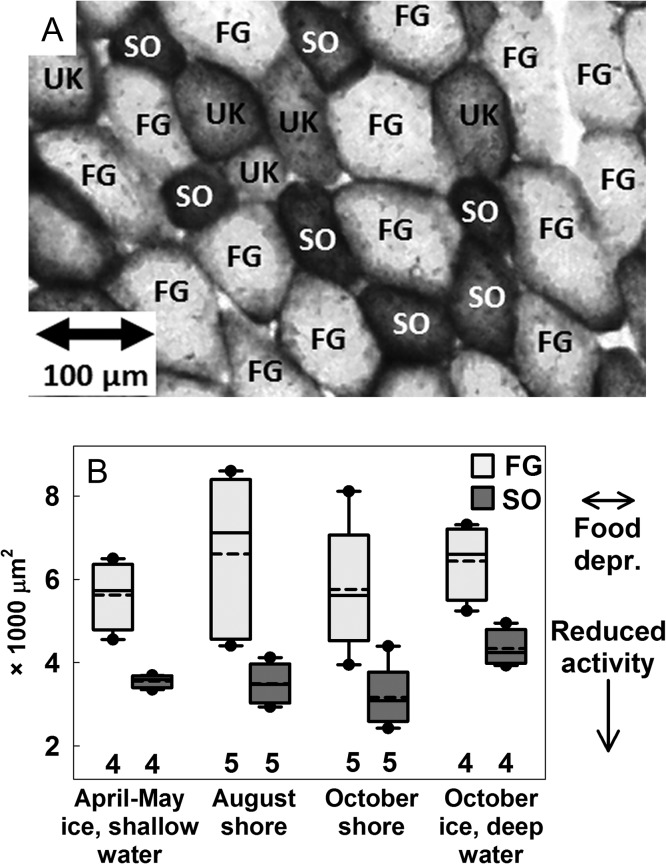
Fiber cross-sectional area in biceps femoris of polar bears. (**A**) Representative cross-section stained for succinic dehydrogenase (×40 magnification). Fast glycolytic (FG) fibers stain light, slow oxidative (SO) fibers stain dark, and fibers of intermediate stain may have been a mixed type (unknown; UK). For (**B**) cell area, dashed and solid lines are mean and median; boxes, bars, and circles are 25th/75th, 10th/90th, 5th/95th percentiles; sample sizes are above *x*-axis. Arrows at the right indicate expected direction of change in response to food deprivation and reduced activity. Data were collected in 2009. Group affected values for SO but not for FG (likelihood ratio for SO [*P* = 0.02] and for FG [*P* = 0.57]). However, no subsequent pairwise comparisons were significant (Table S3).

Type I and IIa MyHC isoforms were detected in all samples (Fig. 5A), based on comparison to brown bear heart muscle (Barrows *et al.*, 2011). The proportion of type I isoforms differed among groups and was lowest for October ice bears (Fig. 5B). This difference was at least partially caused by changes within individuals (Fig. 5C). Cross-sectional area, as measured for a mean (±95% CI) of 96 (±9) FG fibers (range 61–135) and 88 (±10) SO fibers (range 51–104) per sample (Fig. 6A), was similar among groups (Fig. 6B).

### Influence of activity levels on skeletal muscle atrophy

**Figure 7:**
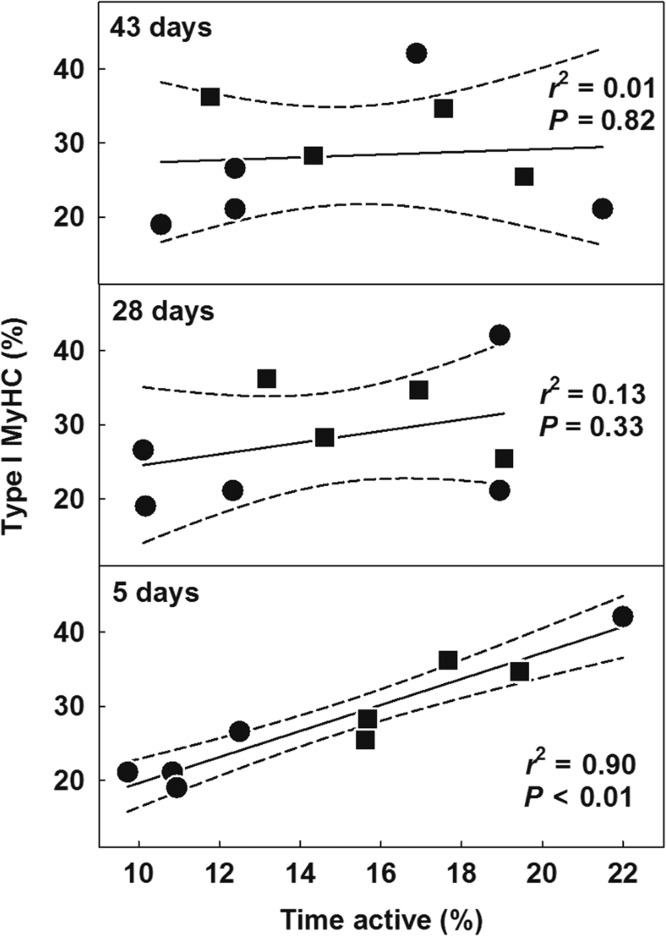
MyHC isoform composition of the biceps femoris in free-ranging polar bears, as predicted by mean activity over the 43 days, 28 days and 5 days prior to sampling. Solid lines show linear regressions (dashed lines show 95% confidence intervals). Symbols represent individual bears: circles show measurements from a sensor that recorded seconds of activity per half hour, and squares show measurements from a sensor that recorded a unitless index of acceleration which were converted to seconds of activity per half hour. Time active was calculated as seconds of activity divided by total seconds every half hour.

Pooling all groups of bears, a total of nine females (mean age ± 95% CI, 10 ± 3 years) had data available on MyHC composition as well as activity and movement rate prior to capture. For these bears, the amount of time spent active was a significant predictor of the expression of type I MyHC isoform if activity was measured over the 5 days prior to capture, but not over the 28 or 43 days (Fig. 7). Movement rate (data not shown) was not a significant predictor of MyHC composition over any of the time periods we tested (5 days, *P* = 0.64, *r*^2^ = 0.03; 28 days, *P* = 0.94, *r*^2^ = 0.00; 43 days, *P* = 0.61, *r*^2^ = 0.04). Of these nine bears, one was sampled in August and eight were sampled in October. For the latter individuals, in the 5 days prior to their capture, the bears on the sea ice were less active (mean % time spent active ± 95% CI, 11 ± 3, *N* = 3) than those on shore (18 ± 3, *N* = 5; Welch *t*-test, *P* = 0.003, d.f. = 5.98).

## Discussion

As predicted, polar bears appeared to experience substantial skeletal muscle atrophy over the winter, the effects of which remained apparent in individuals sampled during April–May. Surprisingly, indices of atrophy did not differ between bears that had hibernated and those that had likely not. There are three potential, non-exclusive explanations. First, individuals that hibernated may have minimized atrophy similar to other bear species. Second, individuals that hibernated may have recovered quickly from their potentially greater atrophy, as suggested by the rapid response of MyHC composition to changes in activity in the 5 days before sampling. Importantly, at the time of sampling in April–May, all individuals may have been successfully hunting for several weeks. The mean sampling date (5 May) was ~8 weeks after females with new cubs typically emerge from dens (13 March; Smith *et al.*, 2013) and ~3 weeks after the peak of parturition season for ringed seals (Smith, 1987). Third, individuals that remained active may have experienced winter food deprivation resembling the complete fast of hibernation, and the associated degree of atrophy. Regardless of the explanation, all bears appeared to experience winter atrophy—and to have recovered during the period of successful hunting in spring and early summer. By August, bears sampled on shore demonstrated no atrophy. In addition, the skeletal muscle of individuals on shore remained essentially unchanged from August to October, likely because these bears had been scavenging whale carcasses (Whiteman, 2014) and were more active than animals on the sea ice during the five days prior to sampling. Ice bears in October exhibited moderate atrophy, consistent with fasting while over deep water during August–October (Whiteman, 2014).

In concert, these data suggest that polar bears may experience larger annual fluctuations in muscle protein than other bear species. It is likely that protein levels for the October shore group represent an annual peak, because these bears had been feeding for ~6 months (Whiteman, 2014). In contrast, protein levels for the April–May group were probably close to the annual minimum as these individuals had likely been food deprived for months during winter, and then only had several weeks of feeding before samples were collected. Although the timeframe of protein recovery after fasting can depend on initial body condition, rate of re-feeding, and other factors, muscle mass may not recover for a month or longer (Therkildsen *et al.*, 2002). The 17% difference in values between the October shore and April–May groups is therefore an estimate of the protein loss between the summer maximum and winter minimum. This estimate implies that seasonal changes for polar bears are high in comparison to black bears which lose ~10% of the protein in their biceps femoris between their probable summer peak (September–December) and winter minimum (March–April; Tinker *et al.*, 1998), and brown bears which lose ~8% between July, likely just earlier than their peak, and February, likely their minimum (Hershey *et al.*, 2008).

The greater fluctuation in muscle protein that we estimate here for polar bears could be a consequence of the April–May group including some bears which experienced extended food deprivation across both the preceding summer and winter (Whiteman, 2014), causing greater protein loss. Also, polar bears almost exclusively consume vertebrate prey (Amstrup, 2003), potentially allowing for more protein accretion than other bear species which may rely more on vegetation and other protein-poor foods (Hilderbrand *et al.*, 1999). All groups in our study had mean protein concentrations (450–541 μg mg^–1^) greater than those reported for black bears (385–430 μg mg^–1^, Tinker *et al.*, 1998) and brown bears (178–194 μg mg^–1^; Hershey *et al.*, 2008), potentially because of differences in their natural history, in laboratory protocols, or in the case of brown bears (Hershey *et al.*, 2008), effects of captivity.

Protein loss is likely related to other changes during atrophy. If water in muscle tissue is conserved while protein decreases, the apparent water content should increase. Indeed, water content was highest for the groups which experienced the greatest protein losses. Tissue water increase is common during food deprivation (McCue, 2010), including in the biceps femoris of black bears during hibernation (Tinker *et al.*, 1998). In contrast, the lack of change in DNA concentration across bear groups is consistent with the hypothesis that as protein (80–90% of dry muscle mass; Zomborszky *et al.*, 1996) was lost, a proportional amount of DNA was degraded (Allen *et al.*, 1995). Muscle cells are multinucleated and if nuclei were added during the hypertrophy of spring and early summer, then lost during winter and late summer atrophy (Gundersen and Bruusgaard, 2008; Brooks and Myburgh, 2014), DNA concentration would remain constant.

It is difficult to distinguish between the consequences of food deprivation and reduced activity because bears may experience both conditions during winter and summer. However, previous studies indicate that some variables respond differently to these influences. Although bear groups did not differ significantly in CN ratio, the lowest mean and median were observed for individuals in April–May, which would be consistent with recently experiencing a long period of winter food deprivation (Koebel *et al.*, 1991). If, instead, inactivity had been a greater influence, CN ratio would be expected to increase (Manini *et al.*, 2007; Marcus *et al.*, 2010).

Fasting, however, should have little effect on the expression of type I MyHC isoforms (Mizunoya *et al.*, 2013). Instead, the decreased expression by October ice bears indicates that summer atrophy was also at least partially caused by reduced activity. These data suggest that in summer, polar bears cannot avoid atrophy similar to black and brown bears in winter hibernation. In our study, during the 5 days prior to sampling, shore bears spent 18% of each day active versus 11% for ice bears, and this difference resulted in a decrease in type I MyHC expression of ~10% for individuals on the ice. In contrast, black and brown bears are active for 50–60% of each day during summer and only 0–2% of each day during winter (Hershey *et al.*, 2008; Laske *et al.*, 2011). Despite this much greater difference in activity than in our study, the unique hibernation physiology of black and brown bears allows them to maintain—or even increase—their type I MyHC expression during winter (Rourke *et al.*, 2006; Hershey *et al.*, 2008).

The reduced expression of type I isoforms for the October ice group appeared to be driven by declining expression within individuals, consistent with previous descriptions of short-term remodeling of muscle phenotype. In lower limb muscles of humans and rats (species not adapted to long periods of inactivity), 4–11 days of unloading reduces type I MyHC expression by 10–28% (Zhou *et al.*, 1995; Stevens *et al.*, 1999). In contrast, despite potentially reduced activity in winter, April–May polar bears did not exhibit a decrease in type I isoform expression, likely because by the time of sampling these individuals had been active for days to weeks. Alternatively, it is possible that polar bears avoid losing type I isoforms in winter despite reduced activity, similar to hibernating brown bears (Hershey *et al.*, 2008), black bears (Rourke *et al.*, 2006), and ground squirrels (*Spermophilus lateralis*; Nowell *et al.*, 2011).

Although the percent of time spent active predicted MyHC composition, hourly movement rate did not, suggesting that it was not indicative of skeletal muscle loading. For ice bears, this may have been because the sea ice was drifting (Durner *et al.*, 2017), causing the movement rate of individuals as inferred from GPS locations to be similar when resting and walking (Whiteman *et al.*, 2015). For shore bears, individuals may have been active but exhibited a low movement rate because they remained near whale carcasses, minimizing spatial displacement. It is unclear why shore bears were more active than ice bears during the 5 days prior to sampling. A possible explanation is that shore bears in the SBS tend to increase their activity after spending more than a month on land (Ware *et al.*, 2017), which would have been the case for many individuals by October.

Other variables provided mixed inferences regarding the effects of reduced activity and food deprivation on muscle condition. Unexpectedly, low activity in the days prior to sampling of October ice bears did not lead to a decrease in cell cross-sectional area. Atrophy may not yet have progressed enough to exhibit this effect, or SO fibers which transitioned to FG fibers shortly before sampling may have retained enough succinic dehydrogenase to still stain as SO (Brown *et al.*, 1976), potentially obscuring declines in area.

Expression of mRNA for several hormones and signaling factors were also not fully consistent with expectations. CK is instrumental in the phosphorylation of myocellular energy stores, however, summer fasting did not lead to a decline in its mRNA in October ice bears. It is possible that polar bears have unusual CK dynamics during summer, similar to ground squirrels, which can also hibernate in winter and which do not show the typical increase in CK mRNA of the plantaris muscle in response to overloading during summer (Choi *et al.*, 2009). Low CK mRNA expression in April–May polar bears was consistent with muscle atrophy during the preceding winter. It is unclear if this was primarily a response to fasting or reduced activity, although in mice, CK mRNA appears to respond more quickly to food deprivation: in the gastrocnemius, expression declined after just 2 days of fasting (Jagoe *et al.*, 2002), but 14 days of disuse via denervation were required before a decline occurred in the soleus (Washabaugh *et al.*, 2001).

Levels of CK and IGF (a stimulator of cell growth and proliferation) suggest that atrophy may have been more severe in 2008 than in 2009, concurrent with the greater physiological stress in 2008 caused by the earlier and more extensive ice melt that year (Whiteman, 2014). Otherwise, MYO, IGF and HIF mRNA expression did not show consistent differences between seasons and habitats. Although these factors predictably respond to inactivity and fasting, the timing of these responses is variable. In humans, mRNA expression for MYO increases after three days of disuse, but remains unchanged for IGF (Gustafsson *et al.*, 2010). It is possible that in our study, differences among groups were obscured by short-term changes in conditions during the days before sampling. In addition, regulatory changes in metabolic pathways could have been post-transcriptional (Philippou *et al.*, 2014), which we would not have detected.

In summary, the changes identified in this study provide a baseline understanding of how polar bear skeletal muscle differs between seasons and habitats, and suggest that food deprivation can have more influence on atrophy than inactivity. Muscle changes primarily caused by inactivity (i.e. reduced type I MyHC expression) appeared to occur, and to be reversed, more quickly than the changes caused by fasting. It is important to note that our results are based only on two years of data collection and that additional variation could occur on longer time scales.

Because sea ice loss (Barnhart *et al.*, 2015) is lengthening the summer fasting period for polar bears at variable rates throughout the Arctic, the risk of muscle atrophy and thus impaired locomotor performance (Hanson *et al.*, 2013) is potentially increasing. Simultaneously, polar bears are being forced to increase their travel in the SBS to remain in high-quality habitat, as sea ice thins and drifts more quickly (Durner *et al.*, 2017). Atrophied muscles fatigue more easily, produce smaller maximum force, and impair balance and coordination (Lopes *et al.*, 1982; Golding *et al.*, 2006; Hanson *et al.*, 2013). Changes in muscle can affect hunting ability; for example, in wolves (*Canis lupus*), predation success peaks at a young age then declines, coincident with a likely peak and subsequent decline of muscle protein and mass (MacNulty *et al.*, 2009). Even small reductions in performance may negatively impact polar bears because they travel extensively in large home ranges (Ferguson *et al.*, 1999) and as large ambush predators, they must match their force exertion to prey body size to ensure hunting efficiency (Williams *et al.*, 2014). We suggest that atrophy related to increased fasting could be a factor contributing to the declines in polar bear survival in the SBS (Regehr *et al.*, 2010; Bromaghin *et al.*, 2015; Rode *et al.*, 2010). Future studies should evaluate locomotion of free-ranging bears, potentially with accelerometers (Ware *et al.*, 2012; Foong *et al.*, 2016), in relation to changes in sea ice extent, composition and drift.

## Supplementary Material

Supplementary Data
