# Supplementary material for: Polar bears experience skeletal muscle atrophy in response to food deprivation and reduced activity in winter and summer
Source: Conserv Physiol. 2017 Aug 9;5(1):cox049. doi: 10.1093/conphys/cox049 (PMC5550809; doi:10.1093/conphys/cox049)

**Table S1. Outlier data points which were removed from statistical analyses.**

| Variable | Value | Group |
| --- | --- | --- |
| Water content | 71.9%  73.4%  79.5% | April–May 2009  April–May 2009  April–May 2009 |
| CK (mRNA) | 7.2^a^  4.0^a^ | October shore 2009  April–May 2009 |
| HIF (mRNA) | 0.0^a^ | October ice 2009 |

^a^Arbitrary units, relative to 18s expression

**Table S2. Statistics from Welch *t*-tests comparing indices of atrophy in biceps femoris muscle of polar bears from two groups in April–May 2009: 1) bears which likely hibernated during the preceding winter, and 2) bears which likely did not hibernate.**

|  | *P* | d.f. |
| --- | --- | --- |
| Protein concentration | 0.73 | 16.3 |
| Percent water | 0.54 | 12.7 |
| DNA concentration | 0.60 | 12.5 |
| CN ratio | 0.99 | 12.5 |
| mRNA (MYO) | 0.39 | 6.6 |
| mRNA (HIF) | 0.63 | 13.3 |
| mRNA (CK) | 0.36 | 8.8 |
| mRNA (IGF) | 0.52 | 14.7 |
| Percent MyHC type I | 0.35 | 15.9 |

**Table S3. Statistics of pairwise comparisons (Welch *t*-test) of variables measured in samples of biceps femoris muscle collected from polar bears in the Beaufort Sea.** Sequential Bonferroni process used to adjust α based on number of comparisons.

| Variable | Group 1 | Group 2 | *P* | d.f. | α |
| --- | --- | --- | --- | --- | --- |
| Protein  concentration | AprMay, 2009 | OctShr, 2009 | 0.001 | 15.65 | 0.008 |
|  | AprMay, 2009 | August, 2009 | 0.010 | 10.03 | 0.010 |
|  | AprMay, 2009 | OctIce, 2009 | 0.055 | 11.00 | 0.013 |
|  | OctShr, 2009 | OctIce, 2009 | 0.237 | 10.29 | 0.017 |
|  | OctIce, 2009 | August, 2009 | 0.463 | 9.00 | 0.025 |
|  | OctShr, 2009 | August, 2009 | 0.636 | 9.26 | 0.050 |
| Percent water | AprMay, 2009 | OctShr, 2009 | 0.005 | 9.95 | 0.008 |
|  | AprMay, 2009 | August, 2009 | 0.058 | 2.94 | 0.010 |
|  | AprMay, 2009 | OctIce, 2009 | 0.105 | 8.44 | 0.013 |
|  | OctShr, 2009 | OctIce, 2009 | 0.185 | 10.93 | 0.017 |
|  | OctIce, 2009 | August, 2009 | 0.270 | 4.74 | 0.025 |
|  | OctShr, 2009 | August, 2009 | 0.976 | 4.94 | 0.050 |
| mRNA (HIF) | AprMay | OctIce | 0.061 | 3.18 | 0.008 |
|  | OctShr | OctIce | 0.100 | 4.03 | 0.010 |
|  | AprMay | OctShr | 0.196 | 13.54 | 0.013 |
|  | AprMay | August | 0.275 | 3.06 | 0.017 |
|  | OctShr | August | 0.406 | 3.36 | 0.025 |
|  | OctIce | August | 0.747 | 4.90 | 0.050 |
| mRNA (IGF) | AprMay, 2009 | OctIce, 2009 | 0.029 | 5.09 | 0.008 |
|  | AprMay, 2009 | OctShr, 2009 | 0.055 | 5.55 | 0.010 |
|  | AprMay, 2009 | August, 2009 | 0.155 | 2.29 | 0.013 |
|  | OctShr, 2009 | August, 2009 | 0.675 | 6.31 | 0.017 |
|  | OctShr, 2009 | OctIce, 2009 | 0.727 | 8.24 | 0.025 |
|  | OctIce, 2009 | August, 2009 | 0.894 | 4.14 | 0.050 |
| mRNA (CK) | AprMay, 2009 | OctIce, 2009 | 0.001 | 6.37 | 0.008 |
|  | OctShr, 2009 | OctIce, 2009 | 0.046 | 8.96 | 0.010 |
|  | AprMay, 2009 | OctShr, 2009 | 0.056 | 5.21 | 0.013 |
|  | AprMay, 2009 | August, 2009 | 0.252 | 2.06 | 0.017 |
|  | OctShr, 2009 | August, 2009 | 0.557 | 2.45 | 0.025 |
|  | OctIce, 2009 | August, 2009 | 0.734 | 2.50 | 0.050 |
| Percent MyHC type I  isoforms | OctShr | OctIce | 0.006 | 8.75 | 0.008 |
|  | AprMay | OctIce | 0.060 | 17.23 | 0.010 |
|  | OctIce | August | 0.077 | 9.58 | 0.013 |
|  | OctShr | August | 0.131 | 10.70 | 0.017 |
|  | AprMay | OctShr | 0.360 | 25.55 | 0.025 |
|  | AprMay | August | 0.736 | 20.34 | 0.050 |
| Cross-sectional area  (SO fibers) | OctShr, 2009 | OctIce, 2009 | 0.023 | 6.56 | 0.008 |
|  | OctIce, 2009 | August, 2009 | 0.030 | 6.85 | 0.010 |
|  | AprMay, 2009 | OctIce, 2009 | 0.031 | 3.73 | 0.013 |
|  | AprMay, 2009 | OctShr, 2009 | 0.304 | 4.42 | 0.017 |
|  | OctShr, 2009 | August, 2009 | 0.433 | 6.88 | 0.025 |
|  | AprMay, 2009 | August, 2009 | 0.786 | 4.95 | 0.050 |

**Fig. S1. Monthly means of sea ice extent in the study area (see box in Fig. 1A).** Ice defined as areas with ≥ 30% during the melt seasons of 2008 and 2009. Data from the National Snow and Ice Center (http://nsidc.org).


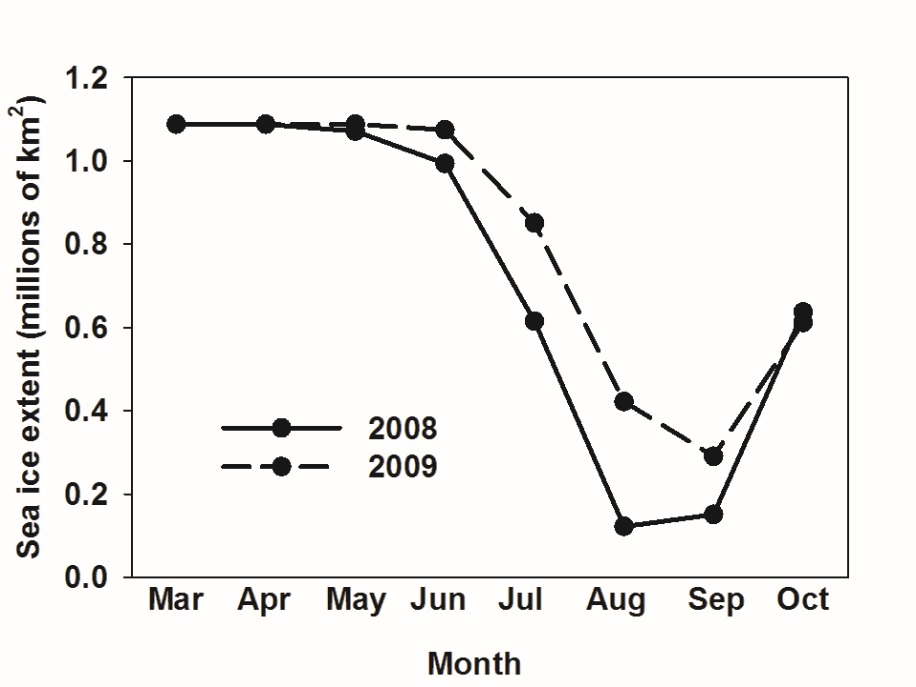

Supplement: Supplementary Data [file whiteman.etal.polarbear.muscle.supp.rev1.docx]
